# Dancing with care: promoting social integration and participation in community activities for older adults

**DOI:** 10.3389/fpubh.2024.1405561

**Published:** 2024-11-14

**Authors:** Jianzeng An, Chun Xia, Jia Xu, Weiwei Li, Jianwen Ding

**Affiliations:** ^1^School of Marxism, Anhui Normal University, Wuhu, China; ^2^School of Educational Science, Anhui Normal University, Wuhu, China

**Keywords:** dancing with care, older adult, social integration, social participation, social capital, community, China

## Abstract

**Background:**

It is common for older adults to move to urban communities after retirement, living with their adult children and caring for grandchildren in China. This impacts their social networks and, consequently, their psychological and physical health. However, research on proactive ways to mitigate the negative effects of social dislocation is lacking. This study examined how dancing with care (DWC), a new form of preventative care, promotes social integration among older adults in Chinese communities, focusing specifically on participants’ experiences related to community engagement, mutual support, volunteer activity, social connections, and advanced learning through their involvement with DWC.

**Methods:**

Semi-structured interviews were conducted with 60 older adults who regularly participated in DWC in communities in southern China.

**Results:**

DWC’s dimensions play a vital role in addressing the social integration of older adults. Through DWC, older adults participate in community activities, engage in mutual support, volunteer in various activities, develop social connections, and learn different things, including the use of advanced technologies.

**Conclusion:**

DWC addresses older people’s social integration by providing opportunities to be involved in the community. It provides a promising path for older adults to actively engage in the revival of social capital within their social networks in the community. This study offers valuable insights for enhancing social involvement for older adults.

## 1 Introduction

The social isolation phenomenon presented in the literature demonstrates that, in China, residents have stopped gathering to chat in the current society (1–3); however, it is unclear when and why this has occurred (4, 5). Researchers have argued that neighbors living on the same floor have become familiar strangers (6–8) because they live in the same apartment building and seldom meet as they enter and exit the apartment building through private garages rather than the common entrances (9, 10). As reported in the literature, alongside declining social capital, elevators have become one of the few places for people to meet (7, 8, 11). Subsequently, neighbors in the same apartment building or surroundings are more familiar with each other’s cars but rarely talk face-to-face (8, 12).

If we analyze the reasons for these social isolation phenomena, we can find from the literature that, for example, marketization and urbanization have changed lifestyles in recent decades, with many people moving to cities and leaving their original social communities (1, 2, 13). The year 2000 was a particular year for China because the central government promoted the development of the community concept and proposed new urban community development plans across the country (14, 15). Before the year 2000, people mostly lived in the same building as their colleagues because it was common for employers to provide apartments (7, 12, 16). After 2000, people moved to self-owned apartments, often in the same community, within a comfortable living environment because they purchased the apartment to ensure a comfortable living environment and better services under the guidelines of urban community development proposed by the government (8, 17, 18).

Older adults retire and move close to family members, such as adult children, to urban communities, where they move into independent living environments, such as independent apartments and houses, to respond to family care needs (19, 20). Simultaneously, older adults, who are also afraid of crime (21), implement safety guardrails around their houses and safety cameras outside their apartment front gate to increase their sense of safety along with the fast development of new technology (22, 23). As a result, research proposed that people in modern society trust digital equipment but less trust people like neighbors around them (24). While doing so, residents isolate themselves (5, 25).

Thus, some questions arise: “How do we reclaim/revive social networks in the urban communities?” (6, 7), or “How do we promote social interaction for older adults?” (7, 8). The answers lie in, for example, encouraging people to integrate into the community and social network through interactive social activities (11) or inspiring older adults to establish a sense of social inclusion based on their preferences and interests (26).

Based on this consideration, the current study aims to explore how Dancing with Care (DWC) promotes social integration among older adults in Chinese communities, focusing specifically on participants’ experiences related to community engagement, mutual support, volunteer activity, social connections, and advanced learning through their involvement with DWC. The current study argues that community-based activities, such as DWC, can promote social integration among older adults by allowing them to participate in community activities in China (27). DWC involves nonprofessional dancing activities, such as folk, modern, street, and Latin dance, usually performed with high-decibel and rhythmic music. While participating in DWC is not age-restricted, older adults after retirement are more likely to join the DWC voluntarily, in particular women, who mostly participate in DWC daily, usually in the evening, after dinner, and dance for around 2 hours (28). Participants may also join the DWC and dance schedules on weekends and during their leisure time. DWC participants usually enjoy their activity in community squares, public spaces, or pathways in the community, and usually, each DWC team has approximately 15 to 30 members, with a few DWC teams of more than 100 members (28, 29). DWC is supported by community staff and often receives financial and structural support from local governments, making it a legally organized and widely accepted form of preventative care in urban Chinese communities. DWC has become a legal, openly organized practice (28, 29). It is, on the one hand, formally organized in communities by DWC team leaders or community staff; on the other hand, it is organically developed by participants or leading participants in the team and forms a DWC team that is noticed by the community. All forms of these openly organized DWC practice get financial and structural support from the local government, and dancing leaders in each self-organized subgroup are formed in the community by residents.

The primary goal of DWC is to promote social inclusion for older adults in community activities, as well as a response to the social isolation many older adults face after moving to urban communities. They are encouraged to engage in mutual support, volunteer in various activities, develop social connections, and learn new skills, including the use of advanced technologies based on DWC. By participating in DWC, older adults can rebuild their social networks, improve their psychological and physical health, and contribute to reviving social capital within their communities. Over the last few decades, DWC was developed by the Chinese Social Welfare Department and echoes concepts described in the United Nations Convention on the Rights of Persons with Disabilities (UN CRPD) (30, 31). The DWC vision in the community was set up to (i) enable the unfettered exercise of choice as to where, with whom, and how they want to live (28, 29); (ii) provide support to people to participate successfully in the community (19); (iii) increase social involvement for older adults (26); and (iv) create a relatively friendly community environment and positive social intervention (26, 29).

Notably, DWC was promoted as a model of preventative care in urban communities by the Chinese government because it was initially designed to improve older people’s health and, in particular, promote early disease prevention and enhance social integration, as exactly what preventative care programs can do in the community (15, 31, 32). However, DWC differs from general preventative care programs due to its flexible participation formation, such as dancing activities based on people’s interests and emotions, in which all DWC participants are taught to dance, share dancing experiences, or teach each other. General preventive care services usually include general physical examinations and selective programs to detect illness, which might lack the power to encourage people’s initiative (25). On the other hand, unlike ordinary preventive care, which is identified as an integral part of disease management regimens and makes people connect preventative care with medical activities (25, 31), DWC participants are not only invited to join dance events but also broaden their social network and become more included in society (32, 33). Non-dance activities, such as shopping, traveling, caring for children, or organizing parties, might also be organized based on DWC team members through daily communication and online chatgroups (34, 35). However, empirical systematic analyses of the impact of DWC on social functions for older adults, such as social inclusion, and health conditions, such as mental health, are lacking. Subsequently, exploring how far DWC impacts older adults’ physiological, emotional, and social functions and mental health is significant.

Subsequently, we offer a conceptual framework of DWC while integrating social capital theory, which proposes DWC dimensions focusing specifically on participants’ experiences related to community engagement, mutual support, volunteer activity, social connections, and the use of advanced learning through their involvement with DWC to promote social integration among older adults in Chinese communities. We conducted semi-structured interviews with 60 older adults who regularly participated in DWC in communities in southern China to investigate how older adults can actively engage in the revival of social capital within their social networks in the community. Furthermore, it offers valuable insights for enhancing the social involvement of community residents.

### 1.1 Literature review

#### 1.1.1 Social capital determines the scope of social integration

People’s involvement in community activities has declined (36).^1^ Fewer residents like to interact, live an organized civic life, pay attention to public topics, and are enthusiastic about public welfare (38). Rather than engaging in community activities, residents prefer being alone at home watching television or digital online entertainment. The decline of social involvement in the community can be seen not only in a single country but also in different cultures and social contexts, such as China and the US (39). Social capital here refers to the interconnections between individuals in society, networks of social relationships, and the resulting mutual benefits and mutual trust among residents (40, 41). The reasons for this phenomenon are complex and signal a decline in social involvement; however, its consequences are clear (42, 43). Putnam (47, 48) views this phenomenon as a loss of social capital. The social capital theory stresses that social networks are closely related to mutual trust and belonging, cooperation, integrated activities in the community (44, 45), and social interactions in daily living, including friendships, sympathy, empathy, and familial relationships (46, 47). Social capital determines the scope of social trust, social cooperation quality, and resident participation level (48).

When people rely solely on themselves, they feel helpless in the community (41). When they have good relationships with neighbors who, in turn, have contact with their neighbors, this expansion could accumulate social capital in the community, which may immediately meet the social needs of individuals. This allows the entire community to create a more comfortable and acceptable living environment (41, 44, 49). Consequently, the community greatly benefits from the cooperation and social integration of individual residents who receive help, sympathy, emotional satisfaction, friendship, and a perception of inclusion (39, 41, 50). Therefore, social capital plays a role in promoting social integration in residents based on the main factors, such as urbanization and suburbanization, generational change, and social connections—that are closely related to enhancing social capital in the community.

#### 1.1.2 Urbanization and suburbanization

As frequent moves lead to weaker community ties, neighborhoods with more frequent changes in residents tend to be less integrated. Residents in unstable communities feel less secure (51) than those in stable communities. Suburbanization indicates greater separation between work and residence and between older people and their peers. Owing to the increasingly sophisticated information technology, in recent times, suburban residents no longer shop on the main streets with acquaintances but in large public shopping malls (52). Moving to join “adult children” in suburban areas and caring for grandchildren has become a common phenomenon for older people. This leads to isolating themselves from their original social network (8). Older people continue to feel alone in apartments and need emotional peer support or “someone to talk to” (53). Urban sprawl destroys the “boundaries” of community residents, and commuting itself strongly negatively affects community participation, decreasing the big, socially connected communities in China.

However, older people have more flexible time after retirement; while some may move to the communities owing to childcare responsibilities, others have leisure time as grandchildren enter kindergarten or primary schools, given the one-child policy in China (3). Women’s retirement age is 55 when they are still actively involved in social networks (54). Some female pensioners take up flexible, temporary jobs based on their interests and to live an abundant life. Their preference for participating in community and volunteer activities, spending more time in entertainment, and visiting friends has increased compared to the past in the last three decades (54). With rare exceptions, most older women are more involved than men in various community activities (55). In addition, older people in China have received more financial support in recent decades from their increased pension levels, comprehensive poverty alleviation policy implementation, and family support. Consequently, older women in the community prefer socially integrated activities that do not contradict their task of supporting their families without worrying about money (56).

#### 1.1.3 Social connection

It has been argued that formal social participation occurs mostly in labor market participation, whereas informal social participation, such as community activities and volunteering, occurs mainly after retirement (57). Formal social participation is modest among the youth, peaks in late-to-middle age, and decreases with retirement. By contrast, informal social participation peaks in young adults and declines as family and social responsibilities increase but increases again after retirement or widowhood (11, 43). Consequently, informal social participation tends to be popular among older adults. Additionally, active older adults can start new social relationships with people in the same community and gain a positive reputation among their peers (58). The closer the connection between older adults in a community, the better they maintain relationships with each other and within the group. If they rarely meet, they may not be able to develop a mutually dependent relationship or may develop a short-lived and weak relationship. Any community life imposes restrictions and demands on organizations; for example, DWC requires a program of meetings and organizational efficiency.

### 1.2 Conceptual framework of DWC

Social integration provides opportunities for involvement in the community, which, in turn, impacts people’s social networks, as well as their psychological and physical health (8, 9, 11). However, scant research has focused on appropriate integrative care activities for older adults to promote social integration in the community (5, 6). The current study found that DWC is a popular care activity that works well to solve this challenge in China. Our research explores how DWC promotes social integration among older adults, focusing specifically on participants’ experiences related to community engagement, mutual support, volunteer activity, social connections, and advanced learning through their involvement with DWC. Subsequently, this study proposes DWC dimensions by integrating social capital theory.

#### 1.2.1 Dimension 1: engagement

Based on social capital theory, people’s norms, beliefs, and values comprise a collective consciousness or shared way of understanding and behaving (57, 58), while society exerts force on individuals through collective consciousness and creates social integration (57, 59). The literature argues that collective consciousness is produced by the actions of and interactions between individuals, producing common sense in a community that holds it together (37, 57, 58). Therefore, a community in society is a product of the individuals’ actions and exerts a coercive social force on individuals (57). Social capital theory proposes that human beings become aware of each other as social beings through their collective consciousness (57). Although collective consciousness permeates the entire society, it has its own characteristics and forms a well-defined reality (60, 61, 77, 78). It is passed down from one generation to the next (62). Scientists argue that collective consciousness is a social psychological form with its own living conditions and development patterns, just like individuals, but in different ways (63, 64). Therefore, behavior is collective if it appeals to a strong and clear collective consciousness in the community, which we explain as engagement (63). People’s engagement in the community can neither be fully promoted through policies or regulations implementation nor entirely be advanced by social force (64, 65). However, it could be protected through the passion generated by the need for social integration of residents (66). We argue that forming this engagement requires less supervision but results in self-organized behavior and self-experience (64). Therefore, people’s experience of participating in community activities in the form of DWC reflects community engagement and promotes their social integration (63). Consequently, DWC’s dimension of participation in the community promotes the development of engagement with social integration.

#### 1.2.2 Dimension 2: mutual support

Social capital theory includes initiatives that promote the understanding of mutual support aimed at reducing social inequalities and exclusion. Mutual support is an individual’s enacted and perceived engagement with social ties, which is important for society’s well-being (57, 60, 67, 77). The literature argues that mutual support is the cooperative behavior of individuals and the solidarity between them (50, 68), and it is the process of incorporating newcomers into society, which explains how well different social groups interact with and accept each other (28, 64). Through mutual support and cooperation, individuals from different groups can share equal access to resources and opportunities or have chances to participate in new social networks. This makes societies more cohesive and promotes understanding and acceptance (69). Mutual support and cooperation are important to prevent premature casualties, diseases, and social exclusion (70). Social networks established through mutual support promote people to stay healthy (71). Relatively, the DWC’s dimension of mutual support reflects social integration with cooperation by improving people’s physical and emotional health conditions. Older adults know each other from DWC and support by sharing information on daily living, shopping, and caring for children. They sometimes voluntarily participate in dance competitions organized by the government to enhance their relationships with DWC peers and increase their cooperation level.

#### 1.2.3 Dimension 3: volunteer activity

Once social integration is strengthened, it enhances the attraction between people, increases their frequency of contact, and enhances the ways and opportunities, such as volunteer activities in the community, for people to form relationships. Social capital theory argues that social integration is, first and foremost, a social capital based on individual organisms (39, 46). Thus, it naturally has a survival ability; it must adapt to the needs and survival mechanisms of people and to their intention to engage in the social community (39, 42, 43). Therefore, social integration outreach could be a common social volunteering activity. The closeness of volunteer activity generated depends primarily on the relationship between the collective consciousness and people’s will to participate in the social activity (38, 42). The more comprehensively the collective consciousness, the more active volunteer activity people have (33). By contrast, a weak collective consciousness will have a weak effect on bringing people into the community (38, 43).

Therefore, we argue that the stronger the willingness of older adults to participate in DWC, the stronger their willingness to engage in more voluntary activity. High fluency in voluntary activity with DWC peers could cause people to have high perceptions of social integration in both participating in DWC teams and joining community activities. The dimension of DWC as a volunteer activity is consistent with this reflection and enhances social integration with community outreach.

#### 1.2.4 Dimension 4: social connection

The power of social cohesion and social communication in a community are affected by social connection (72). People with good social connections produce good benefits in a community (73). Effective communication in the context of social connections could enhance significant social integration development (74). Social connection is explicitly seen as a means to satisfy community needs based on social capital theory (6, 39, 43). For example, older adults move into communities where their adult children live to support their family care needs (46). Older adults’ need for social connections should also be satisfied in communities by participating in community activities, such as DWC. Therefore, DWC offers an opportunity for older adults to mediate the association between social connectivity and social needs. It also helps older adults broaden their social network and accumulate wider social relationships. Thus, the DWC dimension as a social connection simultaneously impacts older adults’ social integration in the community. We have revised the text accordingly.

#### 1.2.5 Dimension 5: advanced learning

Some scholars view social integration as promoting the establishment of social relationships for older adults in the community, which are based on social networks and community activity (37). People who live in the same community or nearby communities have geographical advantages, which are easily reflected in participating in similar activities and establishing social contact or learning experiences and interests with each other (60, 77). For example, older adults who participate in DWC usually share similar geographical living places, while they could establish social relationships (such as friendships) based on family relations and neighborhood. We argue that sharing experiences, learning from each other, and finding common interests through participating in DWC promote the development of social relationships in the community, which react to DWC promotion simultaneously. Furthermore, social relationships easily emerge within a community through mutual learning among older adults as DWC peers (75). For example, older adults’ learning experiences for physical examinations, taking care of the next generations, shopping for groceries, traveling with better plans, and participating in dancing competitions are significant for maintaining friendships. Thus, the DWC dimension of advanced learning impacts social integration by promoting community relationships.

## 2 Method

The analysis included field interview data on the prevalence of DWC activity in communities in the targeted provinces in China, the progress of DWC activity, information on the extent of older women’s access to DWC in their local communities, and whether they received any support by participating in DWC activities. Data on DWC development in the community were collected and reviewed by a research team at Anhui Normal University. The researchers were asked to describe DWC activities in terms of team size, form, organization, staffing, location, and participation. A template was used for data collection to retain all data sources.

In-depth semi-structured interviews were conducted with older adults in 12 communities in Anhui Province, South China. We used snowball sampling in Wuhu to select these communities (76). In Wuhu, officials from the Wuhu Civil Affairs Bureau were asked to recommend three communities that met the following criteria: (a) located within the jurisdiction of the selected city, (b) established for at least 5 years, (c) had official documents supporting DWC activities and practiced DWC activities continuously for more than 1 year, and (d) had at least one nonprofit organization (NPO) joining the implementation of DWC activities, lasted for more than 1 year, and had continuous DWC activities in the past 6 months. After investigating the recommended communities, we asked the community leaders to recommend two or more communities that met the above criteria. The research team interviewed the recommended communities and obtained new recommendations until 12 communities were interviewed.

Sixty respondents (five from each of the 12 communities) were included in the study. The interviewees were older people aged >60 years. We contacted them based on the following inclusion criteria: older than 60 years, lived in the community for at least 9 months in the last year, participated in DWC at least once a week during the past 12 months under normal conditions (not sick, good weather, and available on location), able to clearly express their views, and willing to participate in the research after understanding its purpose. With the support of community staff and NPO leaders in each community, we randomly selected residents who regularly participated in DWC activities via the WeChat group, divided users into multiple WeChat groups, deleted duplicates based on their usernames and avatars, and randomly selected ten candidate residents. One person was randomly selected from the ten selected residents; if they refused to participate in our study, we randomly selected another person from the remaining nine residents. This process was repeated until five residents were selected from each community.

The interviewers were well-trained research assistants (postgraduate students) and our skilled and experienced research team members, who had conducted resident interviews for more than 3 years. We invited nine postgraduate students majoring in public administration as student research assistants to run interviews. Before conducting the formal interview into practice, they were systematically trained and practiced at least five times to ensure that they could conduct the interview smoothly and handle problems that might occur during the interviews. At the same time, during each interview, experienced researchers led the research assistants into the community and communicated with the respondents in advance, informing them that the interview followed the principles of voluntariness and confidentiality and signed the informed consent. The respondents were aged between 60 and 81 years, with an average age of 66.92 years; 48 were women, and 12 were men; the average number of years of participation in DWC was 6.55 years; and 32 self-reported having at least one chronic disease.

Each interview lasted between 35 and 60 min. After obtaining the consent of the interviewee, the research assistants recorded the entire interview process and transcribed the interview record into text materials. The members of this research team reviewed each recording and text. Finally, an original database containing recordings and text was formed, while we also numbered the interview recordings and text in the process. When coding and further analyzing the interviews, the data were randomly distributed, so the interviewer and the person who conducted the data analysis were not the same.

Before conducting the formal interview, we designed a detailed interview question outline based on the research aims and relevant literature (see Appendix 1), and during the interview, we tailored our questions based on the respondents’ interesting responses. The research team developed interview question guidelines based on three levels: primary question content (including three main questions), core question content (including 13 main questions), and extended question content (including six main questions). The interview data were thematically analyzed and coded using a data analysis procedure, which included the preparation and revision of a codebook, training, and evaluation for coders, independent coding by trained coders, and verification and implementation of the coding results. First, following the procedure recommended by Neuendorf (94) and Bengtsson (95), this was based on literature outcomes and our research aim and was conducted by five authors in the research team. Second, we conducted coders’ training and evaluation processes. The coders of this study consisted of two authors (the fourth and fifth authors of this study). We randomly selected five interviews from 60 interviews as training materials (independently), and then the rest of the authors analyzed their coding results. The Holsti coefficients of the two coders were between 0.738 and 0.972. All authors then discussed the tested coding results and gave feedback on the problems of coding. Next, we selected five of the interviews from the remaining 55 interviews, and our two coders conducted the coding again. The team members tested the coding results and found that the Holsti coefficients of these five interviews were between 0.961 and 0.994. Third, the corresponding author selected 25 interviews from the remaining 50 interviews and then randomly selected five from the remaining 25; a total of 30 interviews were included in the formal coding process, which the first coder coded; the corresponding author then randomly selected five interviews from the 25 interviews selected for the first round, plus the remaining 25 interviews from the first round, and handed them over to the second coder for coding. Each coder independently completed the coding of 30 interviews, while among these 30 interviews, both coders coded ten interviews. Other authors calculated their intercoder reliability based on these ten interviews and found that the Holsti coefficient was between 0.964 and 0.996, indicating that the coding process of this study has good intercoder reliability. In the cases when there were discrepancies between coders, we asked the coders to follow our designed coding procedure and coding principles. Then, we organized a discussion meeting again with the participants, all research team members, and three invited experts, who discussed the coding discrepancies, and the consensus reached at the discussion meeting was used as part of the final coding results.

In the coding process, we conducted content analysis coding based on the collected data and conducted relevant coding. During the coding process, no software was used to assist due to financial support limitations. The interview text from each interview was prepared in Chinese, translated into English, and verified by professional language editing services. The study was conducted following the Declaration of Helsinki. The research plan was approved by the University’s Ethics Committee (AHNU-ET2023116). During the implementation phase, we respected the dignity and decision-making rights of each respondent, informed them of the research purpose in advance, and obtained their informed consent before conducting the interviews. Further, we focused on protecting the respondents’ well-being. As the main interviewees were older, the time and location were set according to their preferences to ensure convenience, privacy, and safety. In addition, for 17 interviewees aged over 70 years, community doctors were present in the waiting room to address any possible emergencies. No public health problems were reported during the interviews.

## 3 Results

### 3.1 Community engagement

Older adults’ experience of participating in community activities in the form of DWC reflects community engagement and promotes their social integration. The DWC team usually maintains close connections with community staff and engages in community affairs. For example, they have supported the community by communicating important messages, such as searching for missing persons and dogs and seeking help for people in danger. The DWC participants joined the community to share emotional experiences and to feel included in the community:

I would not say it is a factor in all communities, but I can say they saved my life because Miaomiao [the cat] is a part of my life. I cannot imagine how sad I would have been if she died. It was very cold, and I lost her at night, which was my mistake of letting her go outside. But, the minute I sent my concern to the dancing group, my peers and neighbors helped me find her. I really have someone from whom I can seek emergency support.

Another interviewee mentioned that their brave act of saving a little girl from drowning was broadcast in community videos:

I received a message from the community staff to discuss the organization of our next DWC competition. Her phone rang, and someone shouted from the other side. I spread an urgent message to my DWC team members and went to the riverside with a community staff member. We could not swim, so I took a rope with a bamboo pole to bring the girl up. They broadcast this process, that is, how I met this urgent need. However, you know, I still remember the eyes of that girl; she was really scared.

Community staff have a channel for collecting information when residents need help, and most of this information comes from DWC team managers in the community because they have a “habit” of communicating with community staff about what is going on with older residents. An example illustrates this in a case where an older woman with dementia was lost, wandering in the snow, and found by DWC participants when they were going home after dancing. We found that DWC participants have fast reaction channels in the community chat groups either online or in practice in our interview. When they were eager to spread information and support other members, more participants from different DWC teams in the community reacted quickly. We identify this finding as effective engagement in the community for DWC participants.

We further explore the potential reasons for the existence of such deep engagement in the community for DWC participants and found that some DWC members join in safeguarding the community participants; patrolling helps them connect with other residents who need support. They also spread messages to invite more people to join the DWC and request the residents to support community development. Moreover, DWC team leaders usually negotiate with the community when they need to organize competitions and practice places as the music may be loud, which may result in resident complaints. Therefore, DWC activities encourage members to participate in communities through negotiation.

### 3.2 Mutual support

DWC’s dimension of mutual support reflects social integration and cooperation by improving older adults’ physical and emotional health conditions. Older adults know each other from DWC and support each other by sharing information on daily living, shopping, and caring for children. Older adults were asked about their social interactions and nostalgia levels before and after participating in the DWC activities. Nostalgia plays a dual role in residents’ social participation and integration. It may hinder residents’ social participation because they may view the past more positively and psychologically yearn for it. However, it may cause people to connect new things with the past, arousing their interests and making them willing to participate similarly in society (79). Nostalgia might be a factor that decreases the development of social integration in a new community. One way to reduce the impact of nostalgia is to recognize reality positively. Older adults were asked how enthusiastic they were about their community life. It seems that society has misunderstood them as a silent generation in the community in which they live as older adults maintain their community actively. For example, the respondents reported nostalgia and presence simultaneously after joining DWC in the community. An older female respondent said that the best time of her day was when she danced with partners during DWC activities, leading her to remember when she enjoyed dancing as a young girl. For the DWC participants, happiness is a consequence of sharing an emotional experience and finding themselves meaningful:

… feeling a lack of usefulness in society is bad; I miss the time when I could manage life and make myself useful in everything as I was young. After joining DWC with my wife, I was inspired by the happiness and suddenly found that I could support other team members with many small things in daily life. Of course, fewer older men participate in DWC than women, but I do not care. I was inspired by the feeling of making myself useful and my life meaningful. I am happy.

Dancing felt like enjoying daily and building a new circle of friends who can do things together and support each other, as one participant explained:

My neighbors upstairs danced with me every evening. We felt happy and healthy, as it was obvious that we did not need to visit doctors as often as we did before. I felt an unexpected relaxation… Yes, [dancing] is amazing; it is an enjoyment that we should have. Neighbors were never so familiar before; we dance, shop, and even travel together. Amazing! I cannot recall wanting to travel with neighbors other than my DWC peers. We have each other.

The respondents also reported positive experiences with mutual support. The reasons for this are complex, but the key factors included increased social integration/connection and peer psychological support. Some DWC participants mentioned activities outside the DWC project, such as nurturing the neighborhood’s dogs or scheduling grandchildren’s parties, which strongly impacted their feelings of self-worth. The literature indicates that altruistic daily behaviors not only improve people’s life satisfaction and happiness (80, 81) but also positively impact long-term health (82), and our respondents expressed their desire for mutual help:

They have a chat group to spread information by asking for help and supporting others. It is just like a family chat group, and everyone is so kind that it is hard to refuse…

### 3.3 Volunteer activity

Social integration outreach could be a common social activity of volunteering. The closeness of volunteer activities depends primarily on encouraging older adults to participate in social activities in the community. High fluency in voluntary activity with DWC peers could cause people to have high perceptions of social integration in both participating in DWC teams and joining community activities. Volunteer activities can reflect social integration within the community, which benefits older adults, communities, and even countries (47, 83). Civic connections can make older adults healthy and wise (84, 85), and life without social integration is unimaginable and can cause older people to experience loneliness (86, 87). The positive implications of volunteer activities allow citizens to solve common problems more easily, as recalled by the DWC participants. For example, some of the most impressive volunteer activities occurred during the COVID-19 pandemic and included helping disabled older residents with food supply and medical care service utilization. We found that DWC participants recalled the perception of being included in the social network through volunteer activities they accessed by joining DWC.

Volunteer activities are a lubricant for the community’s wheels of progress. When community members trust each other and interact, the cost of daily business and social interactions is significantly reduced:

…[the number of]” strangers” around us is reducing, people say “hello” because we helped them voluntarily and they say “this is ‘she’ who…” I know they trust me more than they did before, and it feels wonderful.

We found that DWC provides the chance to interact with more people and share experiences and emotions of voluntarily supporting each other. While volunteer activity broadens cognition, cultivates a healthy personality, and promotes good relationships with others (88). People who interact subjectively with others have active connections and maintain character traits that benefit others in the community (89). For example, we recognized a DWC participant who had voluntarily checked for parabola problems on the high floors of community buildings for 4 years during our interview. This DWC participant expressed that he has changed his behavior and believes that more DWC participants can help others do the same by sharing their volunteer experiences to encourage more potential participants to join their DWC team.

Additionally, older adults with abundant volunteering experience can cope with trauma and overcome diseases more effectively. Our study indicates that volunteering improved health and well-being (47):

When I first moved into the community, I was a little worried about the safety of my family, so I did not dare go out at night. Later, I got to know my neighbors who lived downstairs. They took me to the DWC, and I got to know many peers in the community. I also know that the community is safe. Now I am no longer afraid of going out at night. People I know help me when I encounter problems. In addition, my fellow DWC dancers and I distribute brochures to prevent telecom fraud among other community residents. I also learned a lot about preventing telecom fraud and will definitely not be defrauded by online scammers. I never thought that DWC would have benefits. Not only does it make me healthier, but also helps me avoid feeling lonely and afraid.

### 3.4 Social connection

DWC offers an opportunity for older adults to mediate the association between social connectivity and social needs. It also helps older adults broaden their social network and accumulate wider social relationships. A community is directly reflected in the people living in it (89). Participation in DWC further develops a collective sense in which people join, influence each other, and coordinate the group. Thus, DWC combines the characteristics of the communities, forming people’s perception of belonging (34). Consequently, participating in activities such as DWC is associated with developing common relationships in the community, as stated by an interviewee:

The most obvious change is having dinner. I did not notice when my neighbors started ringing my doorbell to share dishes. After that, I did the same to share what I cooked. You know, for a long time, people did not care what others had for dinner.

Thus, the DWC dimension as a social connection simultaneously impacts older adults’ social integration in the community. Similar activities for older adults engaging in DWC are needed for friendships to flourish. However, they must have easy and regular gatherings to connect and maintain friendships. For example, activities organized by a DWC team are of direct significance.

Writing something to friends, such as a card, to celebrate is rare today. People say “young children” do not like it anymore. But, I think we still like this, just that no one cares. I am excited that my friends in the DWC team reminded me of this by sending a Chinese Lunar Year greeting card to me. We kept doing this in the following years, just for happiness.

Social connections created by DWC enhanced the “hands-on” culture in the community which has been reducing in the past decades in China. Older people are still excited about recalling the classic forms of community and social interaction activities, such as cutting paper, cooking, and writing Chinese calligraphy together.

### 3.5 Advanced learning

The DWC dimension of advanced learning impacts social integration by promoting community relationships. We found that learning from others stimulated common preferences within the community. Specific ideas and feelings are derived from this original preference (90). Different types of preferences, such as physical and mental conditions, express both ongoing and temporary characteristics (91). Similar interests and preferences can bring people together, leading them to join similar community activities (14), as expressed by the participants:

No one tells me how to integrate into the group but myself. I can dance when I see others, and I can do it well. It feels like dancing, and the music brings us together. We should have been old friends long before, but just not seen each other for a long time, instead of new peers in the community.

DWC promotes social connection by enhancing common habit formation, which was initially pleasant and enjoyable for older people and which people recognize as their preference. Thus, special behaviors based on preferences are more likely to become habitual and appear uniquely—a certain way of life that, as a habit, makes people feel comfortable and is ultimately indispensable to them, such as DWC. A close social connection may enable people to integrate more socially with assimilation, which could be reflected in honesty and trust (92). Those who have moved from rural to urban areas, especially older adults, have relatively low trust because they do not know the people around them and do not understand their communities. Moreover, they lack confidence (93). When residents have low trust, they may wrongly express their feelings about the social environment (86, 87). The more unfamiliar the action, the more painful, boring, and unwilling people are to do it.

On the contrary, this study found that DWC not only promotes the common preferences and habits of older people but also stimulates them to form common memories of DWC. Habits and memories are related to each other (94). Memory breaks away from habits and sublimates into a special form that exists in people’s cognition (95). Habit returns as an increasingly powerful force (94). Therefore, emphasizing habits and the interaction of memory and habit can encourage people to incline towards conscious or unconscious actions over time (96), such as performing DWC. Consequently, only increasingly smaller or more common stimuli are needed to manifest the action of the DWC. Being accustomed to DWC is equivalent to affirming it, and liking it is consistent with affirming it. We argue that if a preference for DWC habits accompanies the memory of DWC, DWC could be a cultivated memory.

## 4 Discussion

The current study argues that DWC has demonstrated its potential to promote the social involvement of older adults in the community. First, DWC activities offer older adults a chance to have a pool of peers to resort to when they feel lonely and helpless. It provides an opportunity to talk to someone while attenuating physical and emotional pressures through dancing together. DWC provides another dimension of supporting older people in the community compared with other social interventions as it supports older adults in making friends and establishing connections with DWC peers, sharing different social activities such as shopping, sending grandchildren to kindergarten, or cooking together based on dancing networks that established in the primary period. In response to social capital theory’s impact on social integration, DWC has a broadening impact on multiple perspectives of older adults’ social lives.

Second, DWC provides a place and possibility for older adults to engage in social participation rather than older adults’ willingness to engage in social participation, which leads to DWC activities. DWC participants gather to chat, share feelings, support each other, and expand their social networks so that more people are included in the DWC social network. Participation in DWC supports older adults’ sense of contribution to the place they share, such as a community. Their enthusiasm for offering volunteer support in the community proves this argument. Thus, the response to the literature review is that older adults do not change their ideas of living or decrease their enthusiasm for contributing to society. Instead, they become more enthusiastic about community activities as they age. They are also eager to over-participate as a lifestyle choice in public life. The problem that blocked their enthusiasm before was that they could not find a way to express their willingness suitably. Under these circumstances, DWC provides them with a valuable chance, and their enthusiasm for volunteering through DWC activities in the community supports this idea. The older DWC participants found satisfaction in volunteer activities to support their peers and collect information on volunteering shared by peers with similar interests.

Third, the development of new digital technology has enabled flexibility in joining communities from home. However, it has increased social communication anxiety among older adults, who need a community life more than they did before. It is argued that the nature of the community has changed quantitatively from traditional ways such as face-to-face to digital ways of online chat and video activities, which do not need older people to participate in person; moreover, these communities occupy a larger part of our social lives. Currently, many older adults encourage frequent collaborations among their peers. However, casual and pleasant social relationships in the community are interrupted by the new digital forms, and older adults are either unwilling to adapt to this new form because they are not used to it or are unable to accept it owing to their inability to use digital equipment. However, participation in DWC activities has become one of their high-quality choices, which allows them to learn from each other and help keep up-to-date with the high-tech development, such as sharing experiences on using smartphones and software in digital equipment for online shopping, visiting doctors through digital appointments, and checking blood test results online, in a way peers can understand.

Fourth, the emerging trend of DWC in the community has created extensive value that needs to be explored by comparing the findings with existing interventions for older adults, such as the establishment of a community club, similar interesting groups, volunteering mutual support activities, health care support, etc. both in China and globally (97–100). It is necessary to awaken to the new era of diversity and social responsibility to develop community activities meant to enhance social integration for older adults. Older adults’ lives have changed with marketization and urbanization. The current lifestyle encourages older adults to participate in community activities while enhancing their social networks. Modern society calls for older adults to move to these new communities while following their preferences and creating socially integrative memories. DWC paves a new way to enhance our sense of community integration for older people in China. Through DWC, older adults can actively create connective social capital, which requires their willingness and motivation. Nevertheless, natural feelings of relaxation, happiness, involvement, and need are the main attractions of DWC. Singing, walking, dancing, advocating, and participating in activities together through DWC could be an aim, irrespective of ideology or social status. Subsequently, the study further provides valuable insights into enhancing global social involvement, which situates DWC in a broader context and highlights the unique contribution it makes to the field.

## 5 Conclusion

Rapid urbanization has drastically changed people’s lifestyle. Consequently, older adults often move from their original residence to urban communities post-retirement. This affects their existing social network and makes them feel less utilized. DWC is a community activity that has rapidly spread across communities in China and helps older adults come together to dance, connect, volunteer in social activities, and develop their social network. This study conducted semi-structured interviews with older adults participating in community-based DWC activities and evaluated the dimensions in which they benefited from DWC. The findings demonstrate that DWC helps older adults integrate into a new community and feel valued while enhancing their mutual learning opportunities and providing opportunities to participate in volunteer activities. Our study contributes significantly to the literature because it provides evidence of how DWC supports the social integration of older adults. The current study highlights a novel approach that can be followed worldwide to improve older adults’ physical and mental health and well-being.

### 5.1 Limitations

This study, however, has its limitations as, first, it mainly focused on Chinese examples. Future studies should examine the preventative care ideology in other cultural backgrounds with a global perspective. Second, this study used a qualitative method to conduct an in-depth discussion on the effects of DWC on older adults’ social integration in the community. Future research should be conducted using a quantitative design and a highly reliable and valid questionnaire to interview people participating in DWC. It would be better if a longitudinal design survey were used. Combining quantitative and qualitative research will also help us better understand DWC from multiple aspects. Third, this study mainly focused on the social integration of older adults. However, there are also young and middle-aged people participating in DWC. Future research should include more age groups to expand the research scope to DWC. Fourth, the current study does not sufficiently explore the barriers to spreading DWC in different communities or regions. Future research could include reflection on the feasibility, scalability, and potential barriers to the widespread adoption of DWC.

## Data Availability

The datasets generated and analyzed in this study are not publicly available because of respondents’ confidentiality. Anonymized data can be obtained from two sources: from the local government, which provided financial support for this study; and from the corresponding author upon reasonable request and with permission from the School of Marxism, Anhui Normal University, China.

## Footnotes

^1^Wang, Q. (2015). Guangchang Wu: An ethnographic study of dance in public spaces. Unpublished (Master’s thesis). Chinese University of Hong Kong.
